# The involvement of tau in nucleolar transcription and the stress response

**DOI:** 10.1186/s40478-018-0565-6

**Published:** 2018-07-31

**Authors:** Mahmoud B. Maina, Laura J. Bailey, Sherin Wagih, Luca Biasetti, Saskia J. Pollack, James P. Quinn, Julian R. Thorpe, Aidan J. Doherty, Louise C. Serpell

**Affiliations:** 1 School of Life Sciences, University of Sussex, Falmer, Brighton, East Sussex BN1 9QG UK; 2 Department of Human Anatomy, College of Medical Science, Gombe State University, Gombe, Nigeria; 3 Genome Damage and Stability Centre, School of Life Sciences, University of Sussex, Brighton, BN1 9RQ UK

**Keywords:** Tau, Nucleolus, Nucleolar stress, rDNA, Transcription, Tauopathy, Alzheimer’s disease

## Abstract

Tau is known for its pathological role in neurodegenerative diseases, including Alzheimer’s disease (AD) and other tauopathies. Tau is found in many subcellular compartments such as the cytosol and the nucleus. Although its normal role in microtubule binding is well established, its nuclear role is still unclear. Here, we reveal that tau localises to the nucleolus in undifferentiated and differentiated neuroblastoma cells (SHSY5Y), where it associates with TIP5, a key player in heterochromatin stability and ribosomal DNA (rDNA) transcriptional repression. Immunogold labelling on human brain sample confirms the physiological relevance of this finding by showing tau within the nucleolus colocalises with TIP5. Depletion of tau results in an increase in rDNA transcription with an associated decrease in heterochromatin and DNA methylation, suggesting that under normal conditions tau is involved in silencing of the rDNA. Cellular stress induced by glutamate causes nucleolar stress associated with the redistribution of nucleolar non-phosphorylated tau, in a similar manner to fibrillarin, and nuclear upsurge of phosphorylated tau (Thr231) which doesn’t colocalise with fibrillarin or nucleolar tau. This suggests that stress may impact on different nuclear tau species. In addition to involvement in rDNA transcription, nucleolar non-phosphorylated tau also undergoes stress-induced redistribution similar to many nucleolar proteins.

**Electronic supplementary material:**

The online version of this article (10.1186/s40478-018-0565-6) contains supplementary material, which is available to authorized users.

## Background

The microtubule-associated protein, tau, was first described as a protein that promotes and stabilises microtubule assembly [43]. It plays a major role in several neurodegenerative diseases called tauopathies, the most common of which is Alzheimer’s disease (AD). Tau is found in both neuronal and non-neuronal cells, has numerous different isoforms and localises to multiple cellular compartments, indicating that it may play many cellular roles [6]. However, for nearly 30 years, the majority of tau research has focused on its role in microtubule biology (stability/assembly) and the implications associated with tauopathies. In AD, tau becomes hyperphosphorylated and/or truncated and forms paired helical filaments (PHF) that become deposited in neurofibrillary tangles (NFTs) in the cell bodies of neurons. These structures, together with amyloid plaques, constitute the main hallmark of AD. The cellular modifications that accompany the generation of these insoluble, fibrous deposits are believed to play an essential role in neurodegeneration.

A nuclear form of tau has been characterised in several cell lines, primary neurons, mouse brain and human brain tissues (reviewed in [6]. Nuclear tau species are often visualised distributed throughout the nucleus, depending on the protocol, antibody used and stage of differentiation [10, 23]. In neurons, non-phosphorylated tau is mostly seen in the nucleus [42], but can localise to the nucleolus during cellular stress [39]. In neuroblastoma cells, non-phosphorylated tau appear in puncta that localise to the nucleolar organiser region [22]. The nucleolus is the major hub for rRNA gene metabolism. Tau has been found to localise with key nucleolar proteins like nucleolin and upstream binding transcription factor (UBF), as well enhance interactions of RNA-binding proteins like T cell intracellular antigen 1 (TIA1) with ribonucleoproteins, suggesting a role for it in rRNA gene metabolism [4, 37, 41]. Tau has been found to co-localise with the pericentromeric heterochromatin [37], to play a role in its stability [26], and regulate transcription [14]. Tau mutations appear to alter chromosome stability [35], while tau pathology induces chromatin relaxation [11, 14].

Non-phosphorylated tau has been found to translocate to the nucleus playing a role in DNA protection during heat stress [39]. Other reports demonstrate that stress induced by formaldehyde or Aβ42 promotes the nuclear influx of phosphorylated species of tau and this coincides with cellular and DNA damage [24, 25, 31]. These studies suggest that nuclear species of tau may be affected differently depending on the type or severity of the cellular stress. However, it is not clear whether the nuclear phosphorylated tau accumulates in the nucleolus and whether the species of tau localised to the nucleolus behaves like nucleolar proteins, such as nucleophosmin (B3) and fibrillarin (FBL) which become redistributed during nucleolar stress [19]. Nucleolar stress is thought to be an early event in cellular dyshomeostasis, preceding apoptosis and occurs in neurodegeneration [2, 8, 40, 44].

To understand the role of tau on nucleolar function and impact of cellular stress on its nucleolar localisation, here we show that nuclear non-phosphorylated tau localises within the nucleolus in undifferentiated and differentiated human SHSH5Y neuroblastoma cells, where it associates with TIP5, the major subunit of the Nucleolar Remodelling Complex (NoRC) and a key player of heterochromatin stability at constitutive heterochromatin and rDNA [34]. We reveal that tau knockdown leads to an increase in rDNA transcription and associated destabilisation of the heterochromatin indicating that it plays a role in rDNA transcription. Furthermore, glutamate induced stress causes a redistribution of nucleolar tau associated with nucleolar stress indicating that tau behaves like other nucleolar proteins. Immunogold co-labelling electron microscopy analysis of human brain tissue sections shows tau localised with TIP5 in the nucleolus, highlighting the physiological relevance of our findings.

## Methods

### Cell culture

Undifferentiated SHSY5Y neuroblastoma cells were maintained in DMEM/F-12 (Life Technologies, UK), supplemented with 1% (*v*/v) L-glutamine 1% (v/v) penicillin/streptomycin and 10% (v/v) Fetal Calf Serum (FCS). For experiments involving differentiated cells, SHSY5Y cells were incubated for five days in a medium containing 1% FCS supplemented with 10 μM trans-Retinoic acid (Abcam, ab120728), followed by two days incubation with 2 nM brain-derived neurotrophic factor (BDNF) in serum-free media (GF029, Merck Millipore). Cells were treated with 2 mM or 20 mM glutamate (dissolved in DMEM/F-12) or untreated two days post-BDNF incubation.

### siRNA transfection

SHSY5Y cells were maintained for 72 h in Accell SMARTpool siRNA against Tau (Tau siRNA) or non-targeting pool (NT siRNA) (Additional file 1: Table S3) at a concentration of 1.5 μM mixed in Accell siRNA Delivery Media (B-005000-100, Dharmacon).

### Western blotting

SHSY5Y cells treated or untreated with a test compound were fractionated using 1X RIPA (Abcam, ab156034), supplemented with protease (P8340, Sigma) and phosphatase (P0044, Sigma). A total of 10 μg of protein from each sample were loaded to 4-20% Mini-PROTEAN Protein Gels (4568094, BIO-RAD), for SDS-PAGE at 100 V. The proteins were transferred to PVDF membrane (IPVH00010, Merck Millipore) at 100 V, then blocked in blocking buffer (5% (*w*/*v*) milk dissolved in washing buffer (TBS-Tween Tablets solution) (524,753, Merck Millipore), and incubated at 4 °C overnight with the different primary antibodies (Additional file 1: Table S1) diluted in the blocking buffer. The membranes were washed in the wash buffer 5× for 10 min each and probed at RT on a shaker for 1 h in the corresponding secondary antibodies diluted in blocking buffer. The membranes were washed 5× for 10 min each and subsequently developed in the darkroom after incubation in Clarity Western ECL substrate for 1 min (1,705,060, BIO-RAD). For loading control antibodies or sequential analyses of other proteins on the same membrane using other antibodies, the membranes were stripped using Restore™ PLUS Western Blot Stripping Buffer (46,428, Thermofisher Scientific), then blocked, and probed as described above. The blots were scanned at high resolution, and then bands were quantified using Image J software.

### Immunoprecipitation

SHSY5Y cells were fractionated using RIPA supplemented with protease and phosphatase inhibitors and 1.25 units of Benzonase Nuclease (E1014, Sigma), and used at least 2 h afterwards for immunoprecipitation using Dynabeads protein G according to manufacturers protocol (10007D, Life technologies). At the final step, the beads-antibody-antigen complexes were eluted in 30 μL of 50 mM Glycine (pH 2.8) and 15 μL 1× Laemmli Sample Buffer (1,610,747, BIO-RAD), supplemented with 1:10 dilution of 2-Mercaptoethanol (Sigma, M-6250), and boiled at 80 °C for 10 min. The beads were separated from the magnet and supernatant (containing the eluted protein) and used for SDS-PAGE/Western blotting.

### Immunofluorescence labeling

SHSY5Y cells treated or untreated with a test compound, were re-suspended in PBS and spun onto a glass slide at 800 RPM for three min using Cytospin Centrifuge (CellSpin I, Tharmac). Cells were fixed with 4% paraformaldehyde/PBS for 15 min, PBS-washed, permeabilised using 0.5% TritonX-100/PBS for 15 min and PBS-washed. The slides were blocked in blocking buffer [4% BSA/PBS/Tween-20 (0.02%)] for 45 min, incubated with primary antibody for 45 min, PBS-washed three times, incubated in Alexa fluorophore-conjugated corresponding secondary antibody for 45 min. The slides were PBS-washed three times, incubated in 1/1000 DRAQ5 (ab108410, Abcam) diluted in PBS/Tween-20 (0.02%) for 10 min and mounted with coverslips using ProLong Gold Antifade mountant (P36930, Life technologies) or ProLong Gold Antifade mountant with DAPI (P36935, Life technologies). For the labelling of 5-Methylcytosine /(5-mC), cells on the glass slides were fixed with 2.5% PFA/PBS for 30 min at RT, PBS-washed, permeabilised for 1 h at RT with 0.5% Triton X-100/PBS. The cells were next washed in wash buffer [PBS/0.1% Triton X-100 (PBST)] and incubated with 2 N HCl for 30 min at 37 °C to depurinate the DNA, followed by 2 × 5 min wash with 0.1 M borate buffer (pH 8.5). They were then rinsed twice with PBS-T, blocked in blocking buffer (1%BSA/PBS-T) for 1 h at RT, incubated with the primary antibody diluted in the blocking buffer for 2 h at RT and washed three times in PBS-T. Then they were incubated with the corresponding secondary antibody diluted in the blocking buffer for 45 min at RT in the dark and washed three times in PBS-T, then stained with DRAQ5 and mounted.

### Confocal microscopy imaging and analysis

Images were taken using a 100× oil objective of LSM510 Meta confocal microscope mounted on Axiovert200M using pinhole size of 1 Airy unit. All images were collected as Z-stacks for all channels using a step size of 1 μm to allow the analysis of the entire signal in the cells. Subsequently, images were Z-projected to sum all signals and then analysed using image J. Five randomly collected images from each experiment and an average of 150 cells per experiment were subjected to the Image J analysis. For the quantification of nuclear foci/cluster, Image J procedure presented by the light microscopy core facility of Duke University was used [9]. For the quantification of total nuclear fluorescence intensities, the nuclei were first segmented by thresholding using DAPI/DRAQ5 channel, excluding fused nuclei or those at the edges. Subsequently, the multi-measure option on the image J ROI manager was used to measure nuclear fluorescence from all channels in only segmented nuclei. The total corrected nuclear fluorescence (TCNF) was then calculated as TCNF = Integrated Density – (Area of selected cell X Mean fluorescence of background readings [3]. For the quantification of nucleolar nP-Tau and Fibrillarin redistribution, Z-stack images were Z-projected to maximum intensity, before cells positive for the redistribution were counted.

### Immunogold labelling transmission Electron microscopy (TEM)

Brain tissue from the middle frontal gyrus of human brain (see Additional file 1: Table S2) was analysed under local ethics approval and provided by London Neurodegenerative Diseases Brain Bank with informed consent as previously described [1]. The immunogold labelling for these sections and the SHSY5Y cells were performed by minimal, cold fixation and embedding protocols, as previously described using an established method that employs PBS+ buffer for dilution of all immunoreagents and washes [1, 38]. Thin sections were collected onto 300-mesh high transmission hexagonal Nickel grids (Agar Scientific), blocked with normal goat serum (1:10 dilution) for 30 min at RT, single or doubled labelled using antibodies for 12 h at 4 °C. The grids were washed three times with PBS+ for 2 min each, then incubated with appropriate gold particle conjugated secondary antibodies for 1 h at RT (see antibody section and results). The grids were next washed three times for 10 min each in PBS+ and four times for 5 min each in distilled water, dried for 5–10 min and then post-stained in 0.22 μm-filtered 0.5% (*w*/*v*) aqueous uranyl acetate for 1 h in the dark. The grids were finally washed with distilled water five times at 2 min intervals and left to dry for at least 12 h before TEM observation.

### TEM imaging and analysis

JEOL JEM-1400 Transmission Electron Microscope with a Gatan OneView® camera was used to image the grids at 120 V. For colocalisation analysis in the human brain, four nuclei per grid, of medium to large size (> 50% of X8000 magnification view), were randomly selected and imaged at X15000-X20000 magnification. Four grids were taken from each case, accounting for one repeat for the two double immunolabelling cases. In all cases, randomised selection was undertaken by identifying nuclei at low magnification (X5000), then imaging at higher magnification. All images were analysed using Image J. For colocalization analysis on brain sections, each observed 15 nm gold particle, signifying a Tau 1 antigen, was checked for colocalisation with 5 nm gold particles, signifying TIP5 antigens. Our definition of colocalisation is; when the number of one antigen (TIP5 particles) within a 5 0 nm radius of the second antigen (Tau 1) is greater than zero (*n* > 0). Gold particles were included in our analysis if; Tau 1 particles measured between 11≤x≤19 nm and TIP5 particles measured between 1≤x≤9 nm. The method of colocalisation analysis was roughly based on the cross-K function; we used the number of gold particles of the first type at distances shorter than a given distance from a typical particle of the second type divided by the area of the 50 nm inclusion circle [29].

### CellROX green assay

Oxidative stress was measured in treated or untreated SHSY5Y cells using CellROX Green Reagent (C10444, Lifetechnologies UK).

### Nascent RNA and protein synthesis

Nascent RNA and protein synthesis were visualised respectively using Click-iT RNA Alexa Fluor 488 Imaging Kit (C10329, Life technologies) and Click-iT HPG Alexa Fluor 488 Protein Synthesis Assay Kit (C10428, Life technologies) following the manufacturer’s instructions and images were taken using a 100× oil objective of LSM510 Meta confocal microscope mounted on Axiovert200M using pinhole size of 1 AU.

### RNA Extraction and Complementary DNA (cDNA) synthesis

RNA was extracted from SHSY5Y cells treated or untreated using the protocol supplied by Lifetechnologies and subsequently used for cDNA synthesis using the High Capacity cDNA Reverse Transcription Kit (4,368,814, Life technologies, UK).

### Restriction digest for DNA methylation assays

Whole DNA extract from control or Tau knockdown SHSY5Y cells were digested with 2 U/μL of HpaII (R6311, Promega) or MspI (R6401, Promega), or they were mock-digested. T0 region was amplified using specific primers and samples were run on 10% agarose gel for quantitative analysis. For further details, see Additional file 1: Table S4.

### Quantitative polymerase chain reaction (qPCR)

Maxima Probe/ROX qPCR Master Mix (2X) (K0232, Life technologies), 20X TaqMan gene expression assay (Life technologies UK, Table S4) and Nuclease-free water were transferred to a white 96-well semi-skirted PCR plate (I1402–9909-BC, StarLab, UK). A standard curve was prepared using serial dilution of cDNA and qPCR was carried out on all samples using Roche LightCycler 480 II (Roche Diagnostics, Basel, Switzerland). See Additional files for detailed methods.

### Statistical analysis

All data were subjected to *Kolmogorov-Smirnov* (*K-S*) normality test and then students t-test using GraphPad InStat.

## Results

### Tau localises to the nucleolus in undifferentiated and differentiated neuroblastoma (SHSY5Y) cells

**Fig. 1:**
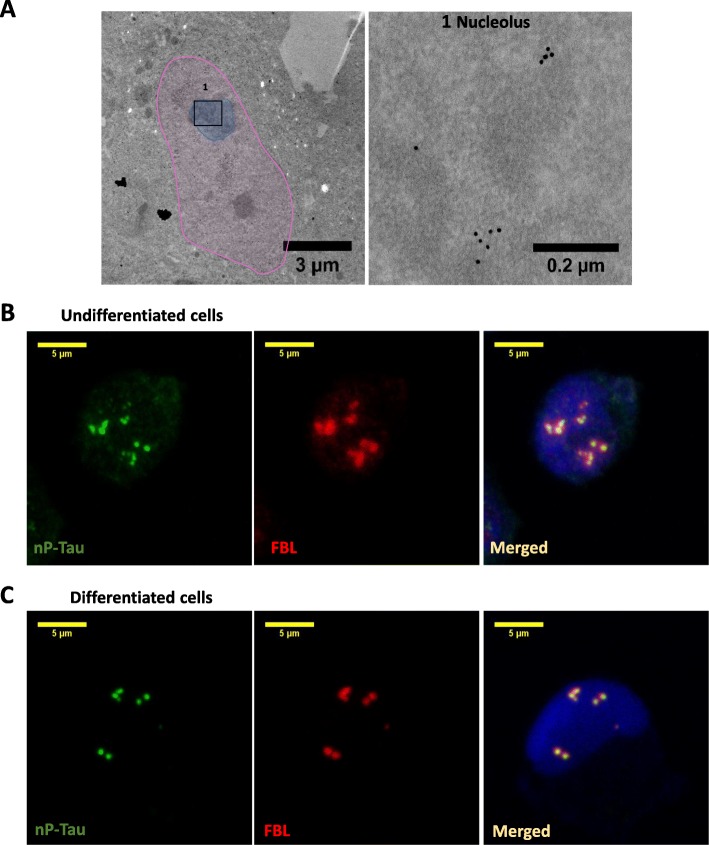
Tau localises to the nucleolus in undifferentiated and differentiated Neuroblastoma (SHSY5Y) cells. **a** Immunogold labelling of undifferentiated cells with T-Tau antibody using 10 nm gold particle-conjugated secondary antibody showed tau gold particles within the Nucleolus (1). Nucleus is highlighted in pink and nucleolus used in second panel is highlighted in blue. The region 1, is identified by a black box. Representative immunofluorescence fluorescence images showing colabelling for nP-Tau and FBL in undifferentiated (**b**) and differentiated (**c**) cells using Tau 1 antibody shows clear punctate distribution of nP-Tau colocalised with fibrillarin (FBL).

There is increasing interest in the non-microtubular roles of the human tau protein. Here we utlised SHSY5Y neuroblastoma cells to investigate tau’s function in the nucleus. This human cell line was chosen as a model system because it expresses human tau at normal levels without the need for overexpression in transfected or transgenic primary neurons. Immunogold electron microscopy using a primary antibody against total tau (henceforth called T-Tau) confirmed the presence of tau in the nucleus localised within the nucleolus in the undifferentiated SHSY5Y cells (Fig. 1a). Nucleolar tau has been traditionally identified using Tau 1 antibody, which identifies tau which is non-phosphorylated on serine 195, 198, 199 and 202 [22], henceforth referred to here as “nP-Tau”. We used an antibody against nP-Tau to investigate the localisation of nucleolar tau using double labelling with fibrillarin (FBL) - a nucleolar marker. Immunofluorescence microscopy showed that nP-Tau was found mainly in the nucleolus co-localised with FBL (Fig. 1b). This colocalisation of nP-Tau with FBL was also confirmed in HeLa cells (Additional file 1: Figure S1A). To explore this association in more neuron-like, non-dividing cells, SHSY5Y cells were differentiated using retinoic acid and brain-derived neurotrophic factor (BDNF). This generates terminally differentiated cells that phenotypical and biochemically resemble neurons, with morphologically distinguishable extended neurites (Additional file 1: Figure S1B) [18]. Immunofluorescence confirmed that nP-Tau colocalises with FBL in differentiated SHSY5Y cells (Fig. 1c).

**Fig. 2:**
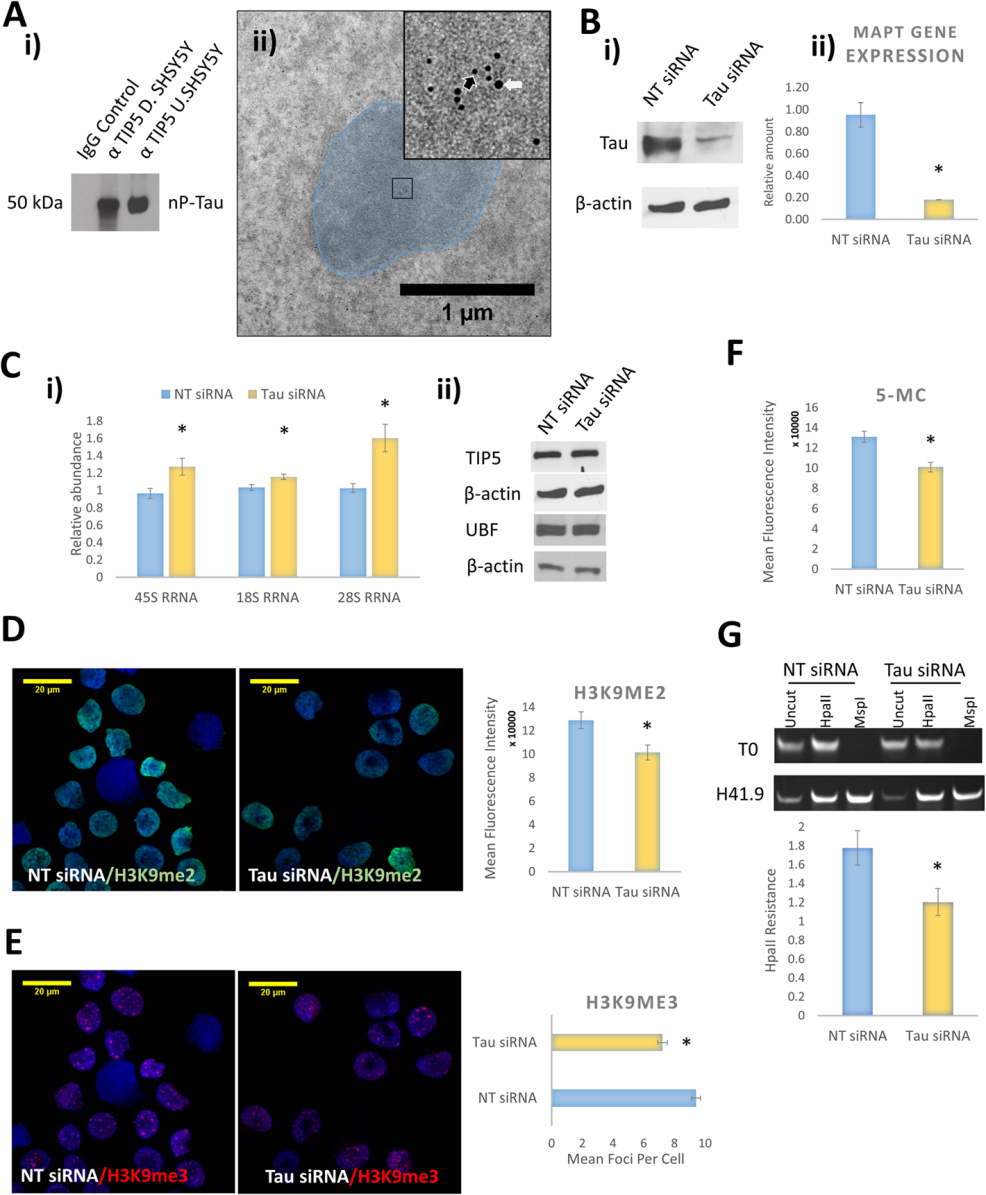
Tau localises with TIP5 and impacts on rDNA transcription and heterochromatin. Immunoprecipitation from whole cell lysates showed that tau associates with TIP5 in both undifferentiated (U.SHSY5Y) and differentiated cells (D.SHSY5Y) (**a**i). Double immunogold labelling revealed that Tau (15 nm) (white arrow) and TIP5 (5 nm) (black arrow) closely associate inside the nucleolus (blue) in SHSY5Y cells (see insert highlighted by black box) (**a**ii). **b** Western blotting (i) and qPCR (ii) to confirm siRNA tau knockdown in undifferentiated SHSY5Y cells. **c**i qPCR on samples from the knockdown cells showed a significant increase in 45S-pre-rRNA synthesis (rDNA transcription), 18S rRNA and 28S rRNA processing. [45S pre-rRNA *P* = 0.017], [18S rRNA *P* = 0.018]; [28S rRNA *P* = 0.0038]. (**c**ii) Western blotting shows that proteins levels of TIP5 and UBF are unchanged in tau knockdown cells. **d** & **e** Representative immunofluorescence fluorescence images showing labelling for H3K9me2/H3K9me3 control and in knockdown cells. Graphs showing quantification from four independent experiments, each with five images and each containing an average of 30 cells. Quantitative immunofluorescence labelling showed that the tau knockdown resulted in a significant reduction in the levels of H3K9me2 [*P* < 0.0001] (D) and number of H3K9me3 foci [P < 0.0001] (**e**). Labelling for 5-Methylcytosine (5-MC) showed that the tau knockdown resulted in a significant reduction in the nuclear levels of 5-mC methylation [P < 0.0001] (**f**). Analysis of HpaII resistance assay showed that tau knockdown reduces the T0 element methylation (**g**). *P < 0.05. Experiment Aii = N2. All other experiments *N* ≥ 4

The localisation of tau to the nucleolus and its role in pericentromeric heterochromatin stability [26], led us to investigate whether it associates with Transcription Termination Factor I-Interacting Protein 5 (TIP5), a protein that localises to the nucleolar and constitutive heterochromatin and crucial for the stability of these domains [36]. Immunoprecipitation (IP) revealed that nP-Tau associates with TIP5 in undifferentiated and differentiated SHSY5Y cells (Fig. 2ai), suggesting a potential role for tau in nucleolar heterochromatin processes. Furthermore, double immunogold labelling using primary antibodies against nP-Tau (Tau1) and TIP5, demonstrated that tau co-localises with TIP5 in the nucleolus (Fig. 2aii). Together, this revealed that in both undifferentiated and differentiated SHSY5Y cells tau associates with TIP5. These results show that nucleolar tau retains its nucleolar localisation and possible function even after differentiation contrary to previous assumptions that its role may be not be needed after differentiation [5].

### Tau knockdown increases rDNA transcription

Depletion of TIP5 has been previously shown to enhance rDNA transcription [34]. To dissect the specific role of tau in the nucleolus, we investigated whether it may play a similar or opposing role to TIP5 in the rDNA transcription regulation. RNA interference using a pool of non-targetting siRNA as control and a pool targetting tau were used to transiently deplete tau, then the functional consequence of its down-regulation was investigated. Undifferentiated SHSY5Y cells were incubated for 72 h with 1.5 μM Accell siRNA resulting in a reproducible and significant loss of tau at both the protein and mRNA levels (Fig. 2b).

Evidence from tau KO mice showed an enhancement of expression of many genes [32] such as an increase in pericentromeric heterochromatin transcription [26]. To explore the consequence of tau knockdown on the nucleolus, rDNA transcription levels were measured. Interestingly, this revealed a significant increase in 45S-pre-rRNA synthesis and processing, indicating an increase in rDNA transcription (Fig. 2ci). Since nucleolar protein levels are important for rDNA transcription and/or processing, the levels of the nucleolar transcription factor, upstream-binding factor (UBF), and TIP5 were measured following tau knockdown. There was no difference between cells treated with tau siRNA and non-targeting siRNA (Fig. 2cii). Overall, this suggests that tau could play a role in transcriptional silencing of the rDNA, similar to TIP5, since its knockdown allowed an increase in transcription of the rDNA.

### Tau knockdown impacts on the integrity of the heterochromatin

Heterochromatin remodelling has been demonstrated to modulate rDNA transcription [21]. TIP5 has been shown to be indispensable for heterochromatin formation and rDNA silencing [13, 34]. Given that we showed an association between tau and TIP5, we speculated that the increase in rDNA transcription may result from the influence of tau on heterochromatin stability similar to TIP5. H3K9me3 and H3K9me2 are impermissive epigenetic markers which are constituents of both nuclear and nucleolar heterochromatin. Depletion of TIP5 has been shown to reduce the levels of H3K9me3 [13, 34]. In untreated SHSY5Y cells, H3K9me2 shows pan-nuclear staining (Fig. 2d), while the H3K9me3 concentrate in foci that indicate constitutive heterochromatin (Fig. 2e). To investigate whether the loss of tau alters the integrity of the heterochromatin we measured the levels and distribution of H3K9me3 and H3K9me2 in tau KO cells and found a decrease in H3K9me3 foci, with an accompanying decrease in the total nuclear intensities of H3K9me2 (Fig. 2d-e), thus showing a loss of heterochromatin following the tau knockdown.

Heterochromatin formation is known to be associated with DNA methylation to provide stability to heterochromatinised genes. To investigate whether tau knockdown also has consequences on DNA methylation, nuclear levels of 5-methylcytosine (5-mC) were measured and found to be significantly reduced following reduction of tau (Fig. 2f). To investigate whether changes in CpG methylation on rDNA are associated with the impact of tau knockdown on rDNA transcription, we measured the level of methylation on the rDNA using restriction digest. Consistent with finding a reduction in global DNA methylation (Fig. 2f), this revealed a significant reduction of the CpG methylation of T0 region of rDNA following the tau knockdown (Fig. 2g). Together, these findings suggest that the increase in rDNA transcription observed following the tau knockdown likely resulted from its impact on the heterochromatin, such that its depletion resulted in heterochromatin loss and transcription permissive environment leading to increased rDNA transcription.

### Nucleolar stress co-occurs with the redistribution of nucleolar nP-tau

**Fig. 3:**
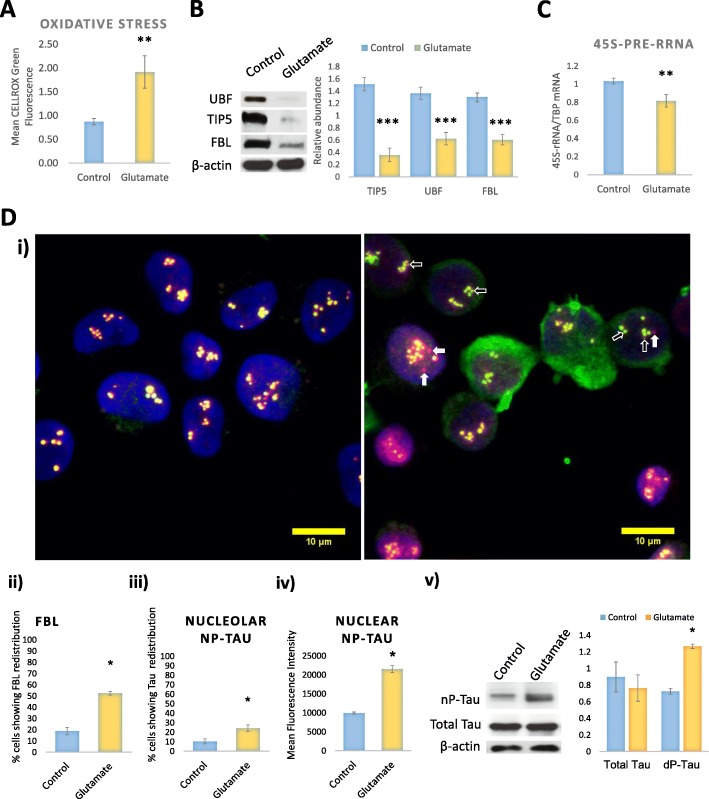
Nucleolar stress co-occurs with the redistribution of nucleolar nP-Tau. **a** Flow cytometry experiments with CellROX Green following 20 mM Glutamate treatment of differentiated SHSY5Y showed oxidative stress [*P* = 0.0013]. **b** Western blotting analysis revealed that the glutamate treatment led to a significant decrease in TIP5, UBF, and FBL. [TIP5 P < 0.0001]; [UBF *P* = 0.0004]; [FBL *P* = 0.0002]. **c** qPCR analysis of rDNA transcription and processing showed that the glutamate incubation resulted in a significant decrease in 45S pre-rRNA synthesis [45S pre-rRNA *P* = 0.008]. **d** Representative immunofluorescence fluorescence images showing labelling for nP-Tau and FBL control and following glutamate treatment (Arrows showing regions in which colocalisation of nP-Tau and FBL is altered by Glutamate treatment). Graphs showing quantification from four independent experiments each with five images and each containing an average of 35 cells. Glutamate administration resulted in redistribution of nucleolar nP-Tau from FBL (blue arrow), as well as FBL redistribution from nP-Tau (white arrows) compared to the control. Analysis of immunofluorescence reveals a significant increase in the number (33%) of cells showing FBL redistribution (**d**ii) [*P* < 0.02]. Quantification revealed that 14% of Glutamate-treated cells showed nucleolar nP-Tau redistribution (**d**iii). [P < 0.02]. Total level of nuclear nP-Tau is increased (**d**iv) [*P* < 0.001]. **d**v Western blotting on whole cell extracts showed a significant increase in nP-Tau, with no changes in T-Tau levels. nP-Tau [P < 0.0001]; T-Tau: [*P* = 0.47]. Intensity normalised to β-actin. Images showing nucleolar tau and FBL in untreated and treated cells were Z-projected for maximum intensity. For all experiments N ≥ 4

Tau’s localisation and functional role are affected by cellular stress and during neurodegeneration. To investigate the impact of cellular stress on nucleolar tau, differentiated SHSY5Y cells were stressed using glutamate. Glutamate has been previously shown to induce toxicity in SHSY5Y cells via a ROS-dependent mechanism [15], and incubation with up to 80 mM glutamate was shown to result in concentration-dependent excitotoxicity at 48 h in both undifferentiated and differentiated SHSY5Y cells [30]. Differentiated cells incubated with 20 mM glutamate for 2 h resulted in significant oxidative stress, compared to the untreated control (Fig. 3a).

The nucleolus is susceptible to cellular stress, causing rapid degradation of nucleolar proteins [7]. To investigate whether the glutamate incubation induces nucleolar stress, the levels of key nucleolar proteins were examined. Western blotting for FBL, UBF and TIP5 revealed a reduction in intensity of the bands for all three proteins and analysis showed a significant decrease in TIP5, FBL and UBF protein levels in glutamate-treated cells compared to controls (Fig. 3b). The rapid decrease in these nucleolar proteins implies that the glutamate treatment directly affects the nucleolus causing its reorganisation. Indeed, different cellular stresses feed into the nucleolus, leading to the regulation of the energy consuming process of ribosome biogenesis through the inhibition of rDNA transcription allowing for the regulation of energy expenditure during stress. To further confirm the presence of nucleolar stress, we measured the levels of 45S pre-rRNA and found that the glutamate treatment led to a decrease (14%) in 45S pre-rRNA, indicative of a reduction in rDNA transcription (Fig. 3c). These findings revealed that the stress induced by glutamate impacts on the nucleolus, causing nucleolar stress, which ultimately results in cell death [40].

Another feature of nucleolar stress is the redistribution of nucleolar proteins, like FBL [19]. We quantified the percentage of cells showing FBL redistribution following the glutamate stress, revealing that 33% of the glutamate-treated cells showed FBL redistribution to the nucleoplasm (Fig. 3di and ii). We then investigated whether nucleolar nP-Tau also undergoes redistribution due to the glutamate stress. Interestingly, although to a lesser extent to the FBL redistribution, approximately 14% of the glutamate-treated cells also showed nucleolar nP-Tau redistribution to the nucleoplasm (Fig. 3di and iii). All cells that showed nucleolar nP-Tau redistribution also exhibited FBL redistribution, while 19% of the cells showed only FBL redistribution and some showed non-punctate, diffuse and decreased FBL staining, indicating the FBL may have been degraded, leaving behind nucleolar nP-Tau (Fig. 3di). A short incubation of cells with lower concentration of glutamate (2 mM) also induced FBL redistribution to a greater extent than nucleolar nP-Tau, but to a lower extent than changes induced by 20 mM glutamate (Additional file 1: Figure S1C). This suggests that there is a dose dependent effect of the glutamate stress on nucleolar tau redistribution and also implies that nucleolar nP-Tau is less susceptible to the stress-induced redistribution/degradation compared to FBL.

Interestingly, while no changes in total levels of tau were observed, the glutamate incubation results in an increase in cellular levels of nP-Tau by western blotting (54%) (Fig. 3dv), which could also be observed by immunofluorescence microscopy (Fig. 3di). This was in-turn associated with its nuclear accumulation (Fig. 3civ), similar to previous studies which suggest a role for this tau species in nuclear protection [39]. Without impacting on total tau levels, a short incubation with 2 mM glutamate also showed a mild increase in the levels of nuclear nP-Tau, further highlighting a concentration-dependent effect (Additional file 1: Figure S1C, E). Its increase in the nucleus may suggest why nucleolar nP-Tau was less affected by the glutamate stress compared to FBL.

### Cellular stress induces the accumulation of phosphorylated tau in the nucleus

**Fig. 4:**
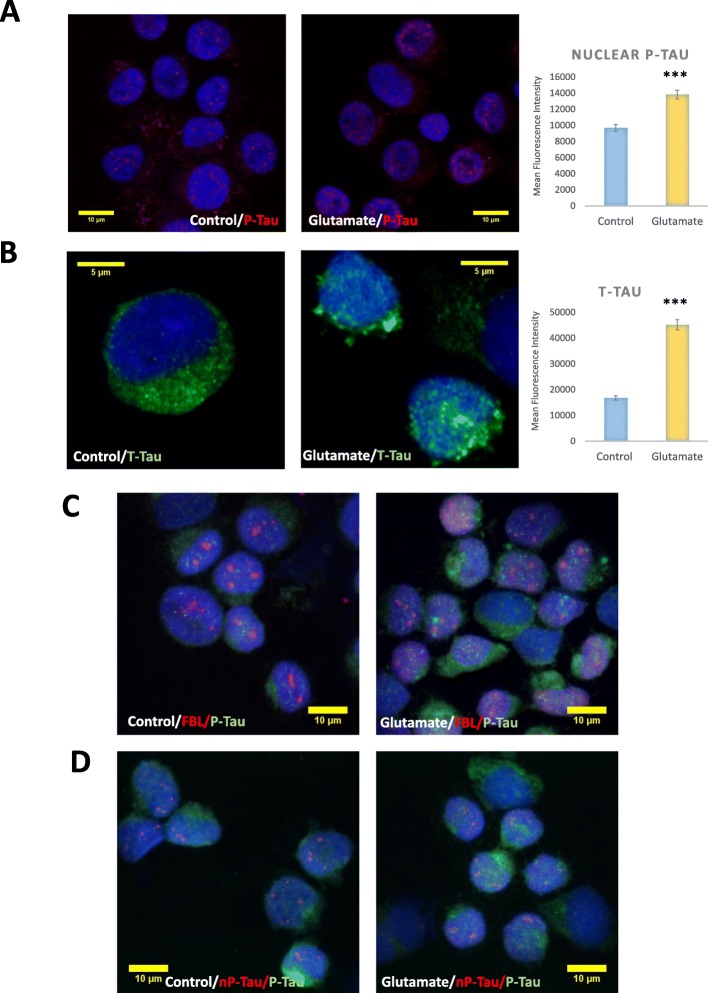
Cellular stress induces nuclear accummulation of P-Tau which does not colocalise with nucleolar markers. Representative immunofluorescence fluorescence images showing labelling for P-Tau and T-Tau control and following glutamate treatment. Graphs show quantification from four independent experiments, each with five images and each containing an average of 40 cells. Immunofluorescence microscopy showed a significant increase in nuclear levels of P-Tau (**a**) and T-Tau (**b**). T-Tau: [P < 0.0001] and P-Tau: [P < 0.0001]. Double labelling revealed that the nuclear P-Tau does not colocalise with FBL (**c**) or nP-Tau (**d**). N ≥ 4

Some studies have demonstrated that cellular stress induces the nuclear influx of phosphorylated species of tau and this coincides with cellular and DNA damage [24, 25, 31]. Effects of glutamate stress on nuclear tau phosphorylation were investigated using immunofluorescence microscopy employing Z stacking to enable direct visualisation of the distribution of nuclear tau with DAPI co-fluorescence to allow unbiased quantification of the signals throughout the entire nuclear volume. Interestingly, this revealed that the glutamate administration also led to an increase in P-Tau (Fig. 4a). Indeed, T-Tau antibody also showed an increase in nuclear tau suggesting an overall increase in tau species in the nucleus (Fig. 4b). A short incubation of cells with 2 mM glutamate also increased both P-Tau and T-Tau nuclear levels, although to lesser extent than with 20 mM glutamate (Additional file 1: Figure S1D). This shows that the glutamate-induced stress results in an increase in different tau species in the nucleus (nP-Tau and P-Tau), which may impact on nuclear function differently [24, 25, 31, 39]. Importantly, given that the response observed following both 2 mM and 20 mM Glutamate treatment occured without any changes in total tau levels (Fig. 3dv and Additional file 1: Figure S1E), suggesting that the changes in the levels of nP-Tau and P-Tau observed is not due to an increase in protein translation.

To investigate whether P-Tau localises to the nucleolus, we examined whether P-Tau colocalises with the nucleolar marker - FBL, or nucleolar nP-Tau. Interestingly, this showed no colocalisation of P-Tau with FBL or with nP-Tau in control and glutamate-treated cells (Fig. 4c-d) suggesting that the P-Tau localises in non-nucleolar nuclear compartment, suggesting distinct roles for nuclear nP-Tau and P-Tau.

Overall, these results revealed that cellular stress impacts on tau species differently, such that some tau may become phosphorylated and accumulate in the nucleus in extra-nucleolar compartments, while nucleolar nP-Tau becomes redistributed. Collectively, these result suggest that under normal conditions, tau plays a role in limiting rDNA transcription, since its depletion leads to an increase in rDNA transcription similar to TIP5. Under conditions of nucleolar stress, nucleolar nP-Tau becomes redistributed similar to other nucleolar proteins such as FBL, nucleophosmin and TIF-IA [17, 20, 27], which ultimately results in cell death [40].

### Nuclear tau in the human brain

**Fig. 5:**
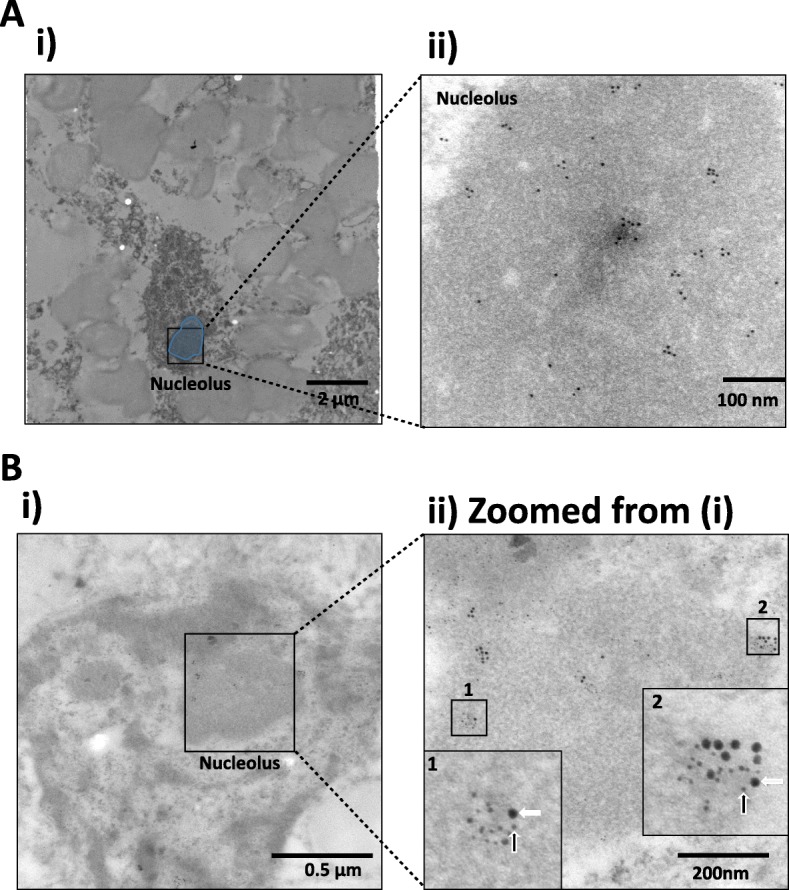
Immunogold electron microscopy to localise tau in the human brain neuronal nucleus. Brain sections labelled with T-Tau (10 nm gold) showed the presence of tau in the (**a**i) nucleus and nucleolus (circle in blue) (**a**ii). Double immunogold labelling for Tau 1 (nP-Tau) (15 nm) (white arrows) and TIP5 (5 nm) (black arrows) showed that they associate in neuronal nucleolus in human brain (**b**i zoomed in **b**ii, see insert for labelling in the nucleolus and nucleolar border). Representative images are shown. Single labelling experiments (**a**) were conducted on sections from two human cases, while double labelling (**b**) was conducted on three cases. For both single and double labelling, four grids were taken from each case, from which four nuclei per grid of medium to large size were randomly selected and imaged

To confirm the presence of nuclear tau in human tissue, we conducted immunogold electron microscopy on middle frontal gyrus tissue sections of human brain. Although tau in the human brain was previously visualised in the nucleolus using immunofluorescence microscopy, because the staining was weak, it was thought that it might not be present in terminally differentiated cells, such as neurons [5]. Under the transmission electron microscope (TEM), heterochromatin appears as electron-dense region, while euchromatin is electron lucent. The nucleolus often appears as darkly stained, granular spherical bodies. Immunogold labelling showed that T-Tau localises in the nucleus, within the nucleolus in the normal human brain (Fig. 5a). Similarly, and in line with our findings in SHSY5Y cells, we observed nP-Tau associates with TIP5 within the nucleolus (Fig. 5b). Co-localisation analysis of gold particles revealed that nP-Tau associates with TIP5 as close as 11 nm apart, and approximately 30% of nuclear nP-Tau is associated with TIP5 within a 50 nm radius. Overall, these findings show a relationship between nP-Tau and TIP5 in both cell models and human brain tissue, suggesting a functional relevance. These results demonstrate the presence of nucleolar tau in the human brain.

## Discussion

Here we reveal a close association between tau and TIP5 in the nucleolus in SHSY5Y cells and in human brain tissue. Based on this association and the widely known role of TIP5 in transcriptional silencing of rDNA, we tested whether nP-Tau plays a role in rDNA transcription. Depletion of tau resulted in increased transcription of 45S-pre-rRNA suggesting a role for nP-Tau in gene silencing and heterochromatin stability. Under conditions of oxidative stress, nucleolar nP-Tau becomes relocalised and the levels of nuclear T-Tau and P-Tau (Thr231) increase in a dose dependent manner.

Tau has been shown to localise with acrocentric chromosomes [22] and heterochromatin in human fibroblasts, lymphoblasts and HeLa cells [37], suggesting it may play a role in heterochromatin regulation. A recent study revealed that tau KO transgenic mice harbour pericentromeric heterochromatin instability, which can be rescued by tau overexpression in the nucleus [26]. Here, we reveal that tau localises to the nucleolus in both SHSY5Y cells and the human brain where it is associated with TIP5. TIP5 has been shown to interact with the nucleolar and constitutive heterochromatin (pericentromeric and telomeric heterochromatin) and plays a vital role in the establishment of these chromatin domains [13, 34]. Here we revealed that depletion of tau led to a reduction in H3K9me3 foci, H3K9me2 nuclear levels and 5-methylcytosine, indicating heterochromatin instability. These results suggest that similar to TIP5, tau may play a role in the heterochromatin complex, such that its knockdown led to heterochromatin loss, likely leading to the increase in rDNA transcription. Previously, tau KO mice also showed that its absence enhances the transcription of several genes [32], including the pericentromeric chromatin [26] and smarce1 gene [12]. Moreover, tau pathology has been found to induce chromatin relaxation and enhance the transcription of many genes suggesting a role for tau in chromatin remodelling [11, 14].

How tau is able to affect chromatin conformation remains unclear. However, we found that tau associates with TIP5 at the perinuclear border, and within the nucleus in the heterochromatin and nucleolus. Such an association may suggest that the heterochromatin and rDNA transcriptional silencing roles of tau may be mediated or facilitated by TIP5 or other chromatin remodellers. TIP5, unlike tau, has different domains that facilitate interaction with chromatin remodellers and the DNA, such as AT-hooks, a C-terminal PHD and a bromodomain [28].

Various cellular stressors are known to induce nucleolar stress, a prominent feature of which is the disruption of the nucleolus and redistribution of nucleolar proteins such as nucleophosmin and FBL to the nucleoplasm or cytoplasm [19, 44]. Redistributed proteins lose their functional role, resulting to cell death [40]. Here we showed that glutamate stress induced nucleolar disruption and redistribution of FBL. However, the striking result observed here is redistribution of nucleolar non-phosphorylated tau. Several studies have located tau in the nucleolus of several cell lines [6, 10], but its disease involvement or impact of cellular stress on its localisation has not been investigated. This study reveals that nucleolar tau also undergoes stress-induced redistribution, similar to other nucleolar proteins, demonstrating a novel involvement of nucleolar tau in nucleolar stress response. Interestingly, several regions of the AD brain have been shown to have a signature of nucleolar stress, associated with the reduction of several nucleolar proteins and nuclear tau [16]. Given the role of tau in AD and many tauopathies, future studies will investigate the changes and contribution of nucleolar tau to the disease pathology. Interestingly, the nuclear P-Tau (Thr231) levels increased in response to stress, but did not colocalise with FBL or with nucleolar nP-Tau. P-Tau Ser396/Ser404 (PHF-1) has been previously shown to localise in the nucleus but not nucleolus, of a patient with presenile dementia with motor neuron disease [33]. On the contrary, it was recently shown that inhibition of transcription with *Actinomycin D* induces the localisation of AT-positive tau (Phospho-Tau (Ser202, Thr205) to the nucleolus in SK-N-BE neuroblastoma cells [10]. This generally suggest that different nuclear tau species may exist and play different roles in the nucleus during a stress response.

## Conclusions

In this study, we establish the presence of nP-Tau in the nucleolus in undifferentiated and differentiated SHSY5Y, HeLa cells and in human brain tissue. We have revealed a novel association for tau and TIP5 in the heterochromatin and nucleolus in SHSY5Y and brain tissue. Although future studies will address the relationship between Tau and TIP5 in heterochromatin stability and rDNA transcription, we postulate that the Tau/TIP5 association may function to stabilise the repressive epigenetic marks on the rDNA and constitutive heterochromatin. This work establishes nP-Tau is a bona fide nucleolar protein which associates with a key member of the NoRC.

## Additional file

Additional file 1: **Figure S1** (A) HeLa cells labelled with nP-Tau and fibrillarin (FBL), showing that they colocalise. (B) SHSY5Y cells before and after differentiation showing extended neurites after differentiation with 5 days treatment retinoic acid followed by 2 days treatment with BDNF. (C) Incubation of cells with 2 mM glutamate led to the redistribution of both nP-Tau and FBL and increased nuclear levels of nP-Tau. (D) Incubation of cells with 2 mM glutamate increased nuclear levels of P-Tau and T-Tau. **Table S1** Antibodies. **Table S2** Brain Tissues. **Table S3** siRNA sequence. **Table S4** List of primers used for ChIP, PCR and qPCR. (DOCX 2016 kb)

## Abbreviations

FBL: Fibrillarin
NoRC: Nucleolar Remodelling Complex
nP-Tau: Non-phosphorylated tau (at positions Ser 195, 198, 199 and 202)
P-Tau: Phosphorylated tau (at position Thr231)
TEM: Transmission electron microscopy
