# Supplementary material for: The involvement of tau in nucleolar transcription and the stress response
Source: Acta Neuropathol Commun. 2018 Jul 31;6:70. doi: 10.1186/s40478-018-0565-6 (PMC6066928; doi:10.1186/s40478-018-0565-6)
Supplement: Supplementary file 1 — Figure S1 (A) HeLa cells labelled with nP-Tau and fibrillarin (FBL), showing that they colocalise. (B) SHSY5Y cells before and after differentiation showing extended neurites after differentiation with 5 days treatment retinoic acid followed by 2 days treatment with BDNF. (C) Incubation of cells with 2 mM glutamate led to the redistribution of both nP-Tau and FBL and increased nuclear levels of nP-Tau. (D) Incubation of cells with 2 mM glutamate increased nuclear levels of P-Tau and T-Tau. Table S1 Antibodies. Table S2 Brain Tissues. Table S3 siRNA sequence. Table S4 List of primers used for ChIP, PCR and qPCR. (DOCX 2016 kb) [file 40478_2018_565_MOESM1_ESM.docx]

**Involvement of Tau in nucleolar transcription and stress response**

**Additional files**

**HeLA Cell Culture**

HeLa cells were grown in RPMI (Life Technologies, UK), supplemented with 1% (v/v) L-glutamate 1% (v/v) penicillin/streptomycin and 10% (v/v) Fetal Calf Serum (FCS).They were maintained at 37°C and 5% CO^2^ atmosphere. For immunofluorescence analysis, cells were collected from culture dishes by trypsinization and cytospun onto glass slide, then processed according to the immunofluorescence protocol.

### Restriction digest for DNA methylation assays

Whole DNA extract from control or Tau knockdown SHSY5Y cells were digested with 2U/μL of HpaII (R6311, Promega) or MspI (R6401, Promega), or they were mock-digested, following which, T0 region was amplified (which has ‘CpG’ sites) using specific primers at Tm 66^o^C (see Table S4) and samples were run on 10% agarose gel for quantitative analysis. For quantification purpose and to avoid loading errors, multiplex PCR was performed, such that, primers against rDNA H41.9 region which does not have ‘CpG’ sites (so will not be cut by HpaII/Msp1), were also run in the same reaction with the T0 primers. This enabled us to normalise the PCR product from the T0 region relative to the H41.9 product that is unaltered by the digestion and then compared the normalised values between control and tau knockdown cells. This way, low level of the HpaII-cut PCR product indicates a reduction in methylation in the HpaII sites of the T0 region which allowed the digestion of the full-length product.

**
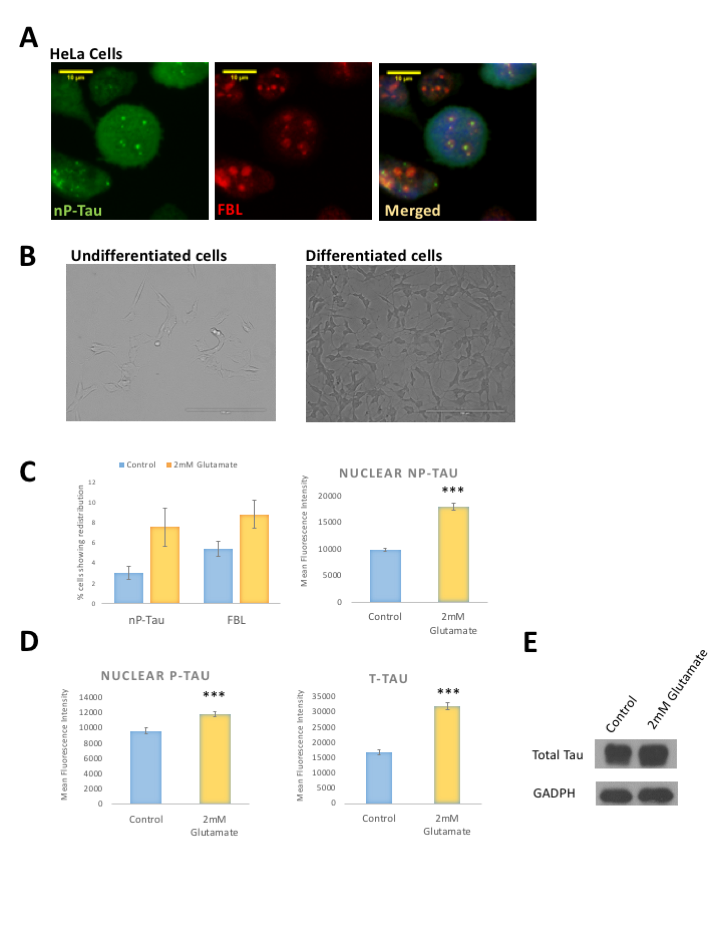
**

**Figure S1.** (*A*) HeLa cells labelled with nP-Tau and fibrillarin (FBL), showing that they colocalise. (*B*) SHSY5Y cells before and after differentiation showing extended neurites after differentiation with 5 days treatment retinoic acid followed by 2 days treatment with BDNF. (*C)* Incubation of cells with 2 mM glutamate led to the redistribution of both nP-Tau and FBL and increased nuclear levels of nP-Tau. (D) Incubation of cells with 2 mM glutamate increased nuclear levels of P-Tau and T-Tau.

**Table S1. Antibodies**

| **Name and Catalogue number** | **Dilution** | **Supplier** |
| --- | --- | --- |
| Rabbit polyclonal anti-TAU antibody (SAB4501831) | IF: 1/100, TEM IG: 1/10 WB: 1:1000 | Sigma-Aldrich |
| Mouse-Phosphor-Tau (Thr231) Antibody (MN1040) | IF: 1/50 | Thermo Fisher Scientific |
| Rabbit Anti-Tau antibody (phospho T231) (EPR2488) | IF: 1/100, WB: 1:2000 | Abcam |
| Mouse Anti-Tau 1 antibody (MAB3420) | IF: 1/200, TEM IG: 1/10, WB: 1/1000, IP: 1/40 | Millipore |
| Rabbit Anti-trimethyl-Histone H3 (Lys9) antibody (07-442) | IF:1/200 | Millipore |
| Mouse Anti-Histone H3 (di methyl K9) antibody [ab1220] | 1/200 | Abcam |
| Rabbit Anti-UBF antibody (H-300) sc-9131 | IF:1/200; WB:1/1000 | Santa Cruz Biotechnology, Inc |
| Rabbit Anti-TIP5 Polyclonal Antibody (49-1037) | WB:1/200, TEM IG:1/50, IP: 1/40 | Life technologies |
| Mouse Anti- 5-methylcytosine (5-mC) monoclonal antibody 33D3 (C15200081-100), | IF: 1/500 | Diagenode |
| Rabbit- Anti-EIF2S1 (phospho S51) antibody [E90] (ab32157) | IF: 1/200 | Abcam |
| Mouse Monoclonal Anti-β-Actin antibody (**A5316**) | WB:1/5000 | Sigma-Aldrich |
| Mouse Monoclonal Anti-Gapdh, Clone GAPDH-71.1 (G8795) | 1/5000 | Sigma-Aldrich |
| Normal mouse IgG (sc-2025) | IP: 1/40 | Santa Cruz Biotechnology, Inc |
| Alexa Fluor® 555 Goat Anti-Mouse IgG (H+L) (A31622) | 1/500 | Invitrogen |
| Alexa Fluor® 555 Goat Anti-Rabbit IgG (H+L) (A31630) | 1/500 | Invitrogen |
| Alexa Fluor® 488 Goat Anti-Mouse IgG (H+L) | 1/500 | Invitrogen |
| Alexa Fluor® 488 Goat Anti-Rabbit IgG (H+L) (A31628) | 1/500 | Invitrogen |
| 10 nm Gold-particle conjugated Goat anti-Rabbit IgG | 1:10 | British BioCell |
| 15 nm Gold-particle conjugated Goat anti-Mouse IgG | 1:10 | British BioCell |
| 5 nm Gold-particle conjugated Goat anti-Rabbit IgG | 1:10 | British BioCell |
| Goat Anti-Rabbit IgG H&L (HRP) (ab6721) | 1/5000 | Abcam |
| Anti-mouse IgG, HRP-linked Antibody (7076) | 1/1000 | Cell Signalling |
| Mouse TrueBlot® ULTRA: Anti-Mouse Ig HRP | 1/1000 | Rockland Immunochemicals Inc. |

Key: IF – immunofluorescence, IP – Immunoprecipitation, TEM IG – Immunogold Transmission Electron Microscopy, WB – Western blotting

**Table S2. Brain Tissues**

| **Case** | **Age** | **Sex** |
| --- | --- | --- |
| Control 1 | 80 | F |
| Control 2 | 66 | M |

**Table S3: siRNA sequence**

| siRNA Target name | Target sequence 1 | Target sequence 2 | Target sequence 3 | Target sequence 4 |
| --- | --- | --- | --- | --- |
| Non-targetting Pool **(**D-001910-10-05**)** | UGGUUUACAUGUCGACUAA | UGGUUUACAUGUUUUCUGA | UGGUUUACAUGUUUUCCUA | UGGUUUACAUGUUGUGUGA |
| Human MAPT – Tau (E-012488-00-0005) | UGGUGAACCUCCAAAAUCA | CUUGCAAGUCCCAUGAUUU | UUGUGAUCUUAAAUGAGGA | UUAUUGAGUUCUGAAGGUU |

**Table S4: List of primers used for ChIP, PCR and qPCR**

| **Primer name** | **Forward sequence** | **Reverse sequence** |
| --- | --- | --- |
| T0 | GCTCCCCGGCCCGGCGCT | CCATCGCAGCCACACACG |
| H41.9 | CCGTGGGTTGTCTTCTGACT | AAGCGAAACCGTGAGTCG |
| RNA18S5 | TaqMan Assay (Lifetechnologies) | assay ID; Hs03928985_g1 |
| RNA28S5 | Taqman Assay (Lifetechnologies) | assay ID; Hs03654441_s1 |
| TBP | Taqman Assay (Lifetechnologies) | assay ID; Hs00427620_m1 |
| MAPT - Tau | Taqman Assay (Lifetechnologies) | assay ID; Hs00902194_m1 |
| Β-actin (ACTB) | Taqman Assay (Lifetechnologies) | assay ID; Hs01060665_g1 |

| Name | Forward | Reverse | probe |
| --- | --- | --- | --- |
| RNA45S | CACCCTCGGTGAGAAAAG | CTACCATAACGGAGGCAG | CTTCTCTAGCGATCTGAGAGGCGTGCC |
